# Cytomegalovirus induces abnormal chondrogenesis and osteogenesis during embryonic mandibular development

**DOI:** 10.1186/1471-213X-8-33

**Published:** 2008-03-27

**Authors:** Tina Jaskoll, George Abichaker, Parish P Sedghizadeh, Pablo Bringas, Michael Melnick

**Affiliations:** 1 Laboratory for Developmental Genetics, USC, Los Angeles, CA, USA; 2 Oral and Maxillofacial Pathology, Division of Diagnostic Sciences, USC, Los Angeles, CA, USA; 3 Center for Craniofacial and Molecular Biology, USC, Los Angeles, CA, USA

## Abstract

**Background:**

Human clinical studies and mouse models clearly demonstrate that cytomegalovirus (CMV) disrupts normal organ and tissue development. Although CMV is one of the most common causes of major birth defects in humans, little is presently known about the mechanism(s) underlying CMV-induced congenital malformations. Our prior studies have demonstrated that CMV infection of first branchial arch derivatives (salivary glands and teeth) induced severely abnormal phenotypes and that CMV has a particular tropism for neural crest-derived mesenchyme (NCM). Since early embryos are barely susceptible to CMV infection, and the extant evidence suggests that the differentiation program needs to be well underway for embryonic tissues to be susceptible to viral infection and viral-induced pathology, the aim of this study was to determine if first branchial arch NCM cells are susceptible to mCMV infection prior to differentiation of NCM derivatives.

**Results:**

E11 mouse mandibular processes (MANs) were infected with mouse CMV (mCMV) for up to 16 days *in vitr*o. mCMV infection of undifferentiated embryonic mouse MANs induced micrognathia consequent to decreased Meckel's cartilage chondrogenesis and mandibular osteogenesis. Specifically, mCMV infection resulted in aberrant stromal cellularity, a smaller, misshapen Meckel's cartilage, and mandibular bone and condylar dysmorphogenesis. Analysis of viral distribution indicates that mCMV primarily infects NCM cells and derivatives. Initial localization studies indicate that mCMV infection changed the cell-specific expression of FN, NF-κB2, RelA, RelB, and Shh and Smad7 proteins.

**Conclusion:**

Our results indicate that mCMV dysregulation of key signaling pathways in primarily NCM cells and their derivatives severely disrupts mandibular morphogenesis and skeletogenesis. The pathogenesis appears to be centered around the canonical and noncanonical NF-κB pathways, and there is unusual juxtaposition of abnormal stromal cells and surrounding matrix. Moreover, since it is critically important that signaling molecules are expressed in appropriate cell populations during development, the aberrant localization of components of relevant signaling pathways may reveal the pathogenic mechanism underlying mandibular malformations.

## Background

Human clinical studies and mouse models clearly demonstrate that cytomegalovirus (CMV) disrupts normal organ and tissue development. It is established that about 2% of live born infants are congenitally infected with active CMV, making CMV one of the most common causes of major birth defects in humans [1,2]. CMV, an enveloped, double-stranded DNA betaherpesvirus, is species-specific and has a slow replication cycle. In congenitally-infected newborns, CMV establishes a long-lasting persistence; active CMV infection in children can last for months and even years after birth before termination of productive infection and establishment of latency [3]. Presently, little is known about the mechanism(s) underlying CMV-induced congenital malformations.

Mouse CMV (mCMV) has many features in common with human CMV (hCMV). Thus, the mouse model has been widely employed for studying the pathogenesis associated with acute, latent, and recurrent infections [4]. CMV infection of embryonic development induces substantial fetal loss, fetal growth retardation, and fetal dysmorphogenesis, particularly of the craniofacial complex (brain and branchial arches) [5-8]. Importantly, Tsutsui [9] found that viral-antigen positive cells were abundant in the mesenchyme of the oral and nasal cavities, and in the mesenchyme of the brain, postulating that mesenchymal infection is the critical step in disrupting organogenesis. If so, oral-facial organogenesis, which is highly dependent on mesenchymal integrity and epithelial-mesenchymal interactions, would be particularly vulnerable to CMV infection. Recent studies in our laboratory demonstrate that first branchial arch derivatives (submandibular salivary glands and teeth) are vulnerable to CMV infection during critical stages of their organogenesis, and that CMV has a particular tropism for neural crest-derived mesenchyme (NCM) [10,11].

Branchial arch formation and differentiation is the *sine qua non *of proper oral-facial development. Branchial arches form as paired mesodermal thickenings in the lateral and ventrolateral pharyngeal walls of the early embryo (E8.5 in mice). Cranial neural crest cells migrate ventrally into the primitive arches from the caudal regions of the developing brain [12-14]. With proliferation of the NCM, the well-defined pairs of branchial arches become visible externally. Of particular importance to oral-facial development, is the first branchial arch which gives rise to the maxilla, palate, teeth, mandible, salivary glands, and the anterior two-thirds of the tongue. The first branchial arch develops as two processes, the smaller maxillary process and the larger mandibular process. The mandibular process (MAN) of the first branchial arch gives rise to the lower jaw. The paired MANs merge with one another at approximately E9 in mice, shortly after they become externally apparent. Cranial neural crest cells generate the majority of MAN mesenchymal cells which differentiate into a wide variety of derivatives, including cartilages, bones, connective tissues, tooth papilla and smooth muscles [12-14].

MAN development is dependent on the presence of Meckel's cartilage which serves as a template for mandibular bone formation, as well as contributing to part of the mandibular bone [15-18]. Meckel's cartilage formation is initiated by the condensation of neural crest-derived prechondrogenic cells, which differentiate into chondrocytes and gives rise to the characteristic rod-shaped cartilage. The cartilage grows anteriorly and posteriorly to develop into a "wish-bone-like" structure, with NCM-derived prechondrocytes being found at the chondrogenic front. Although cranial neural crest-derived and non neural crest-derived cells contribute to Meckel's cartilage [13,19], it has become apparent that neural crest-derived cells play the primary role in Meckel's cartilage initiation, growth and chondrogenesis (see review [20]).

Meckel's cartilage serves as the primordium of the mandible and middle ear ossicles which differentiate from 3 distinct regions: the distal region which contributes to mandibular bone development and undergoes endochondral-like ossification to give rise to the mandibular symphysis; the middle region which serves as the template for mandibular membranous bone formation; and the most proximal region which undergoes endochondral ossification to give rise to the malleus and incus [15,21-23]. Thus, the mandibular bone, a cranial NCM derivative [13], is formed by both endochondral ossification of Meckel's cartilage [18,24,25] and intramembranous ossification around Meckel's cartilage [21,22]. The coronoid, condylar and angular cartilages, classified as secondary cartilages, undergo endochondral ossification in conjunction with mandibular ossification [26].

Active mCMV infection of embryonic mouse Canalicular stage submandibular salivary glands or Cap stage mandibular first molars *in vitro *induces severely abnormal salivary gland and tooth phenotypes [10,11]. MCMV-infected salivary glands are significantly smaller and exhibit atypical ductal epithelial hyperplasia, apparent epithelial-mesenchymal transformation, and oncocytic-like stromal cell metaplasia; mCMV infection induces developmentally-delayed, dysmorphic molars characterized by shallow, broad and misshapen cusps, infected and affected dental papilla mesenchyme, poorly differentiated odontoblasts and ameloblasts, and diminished enamel and dentin matrix. Genomic and protein analyses indicate that the mCMV-induced pathogenesis is primarily mediated through NF-κB signaling and that there appears to be an unusual interaction between abnormal NCM cells and corresponding extracellular matrix (ECM) [10,11].

Early embryos are barely susceptible to CMV infection, and the extant evidence suggests that the differentiation program needs to be well underway for embryonic tissues to be susceptible to viral infection and viral-induced pathology (see review, [9]). Still, it is unclear whether first branchial arch mesenchymal cells are susceptible to mCMV infection prior to differentiation of skeletal elements. To address this, we used a chemically-defined organ culture system to investigate the effect of mCMV infection on embryonic MAN morphogenesis and differentiation of skeletal elements. Active infection of embryonic day 11 (E11) predifferentiated MANs results in micrognathia consequent to decreased Meckel's cartilage chondrogenesis and mandibular osteogenesis. We also demonstrate that mCMV infection induced changes in important components of relevant signal transduction pathways.

## Results

**Figure 1:**
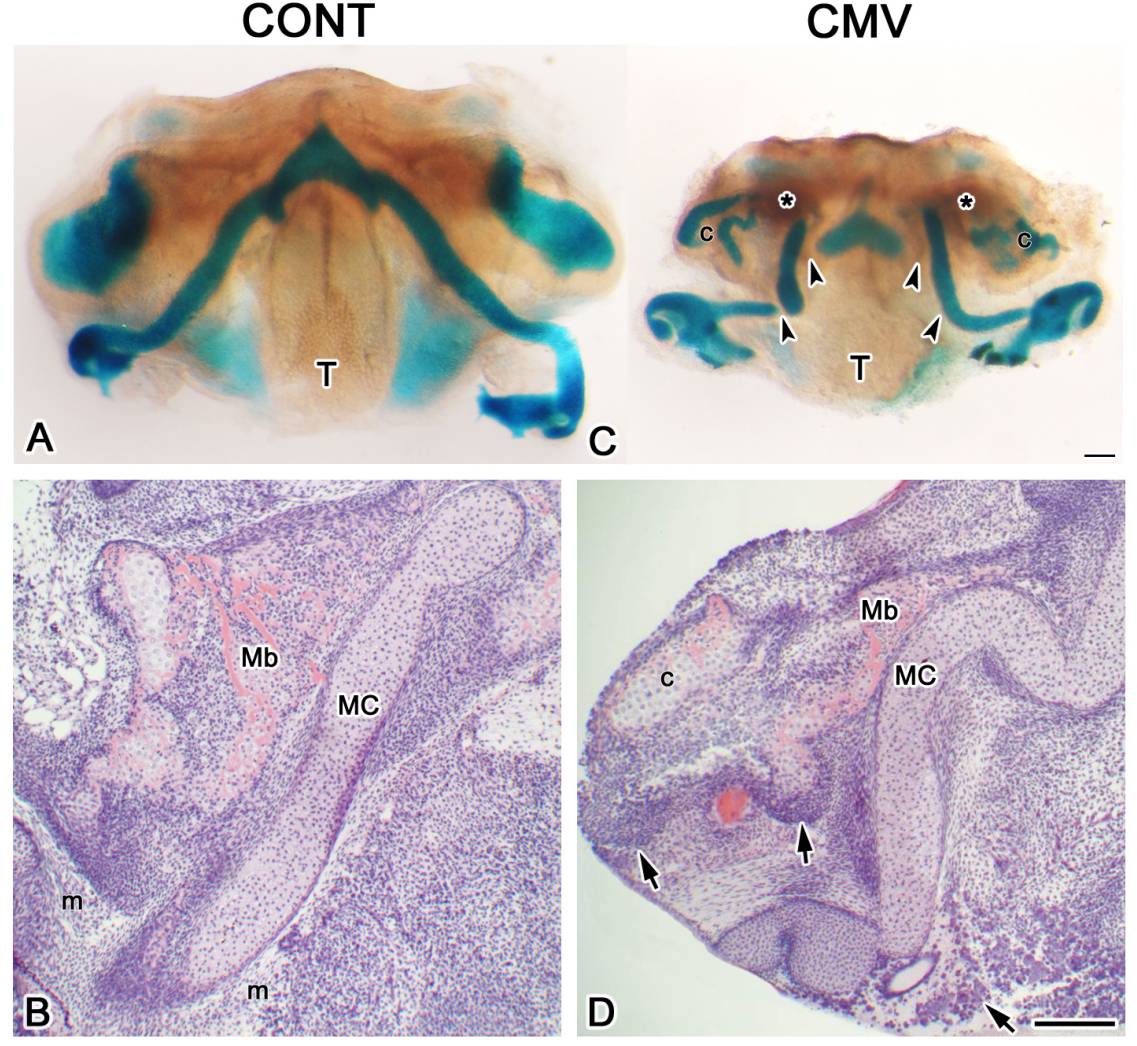
mCMV-induced micrognathia and abnormal skeletogenesis in mCMV-infected E11 + 10 mandibular processes. A, C. Alcian blue-stained cartilages and alizarin red-stained bone in control (A) and mCMV-infected (C) MANs. mCMV-infected MANs are significantly smaller and exhibit a misshapen Meckel's cartilage with decreased chondrogenesis (arrowheads), reduced mandibular bone osteogenesis (*) and aberrant condylar (c) chondrogenesis, as well as tongue (T) hypoplasia. B, D. Histological analysis. In controls (B), the characteristic rod-shaped Meckel's cartilage (MC) and adjacent ossifying mandibular bone (Mb) are surrounded by mesenchyme (m) which is primarily derived from cranial neural crest. mCMV-infected MAN (D) skeletal components are severely hypoplastic, characterized by a bent and developmentally-delayed Meckel's cartilage, reduced mandibular bone ossification and abnormal condylar (c) formation. Note the presence of clusters of large, pleiomorphic and basophilic stromal cells (arrows) in the periphery and surrounding Meckel's cartilage, mandibular bone and condyle. Bar: A, C: 100 μm; B, D: 50 μm.

**Figure 2:**
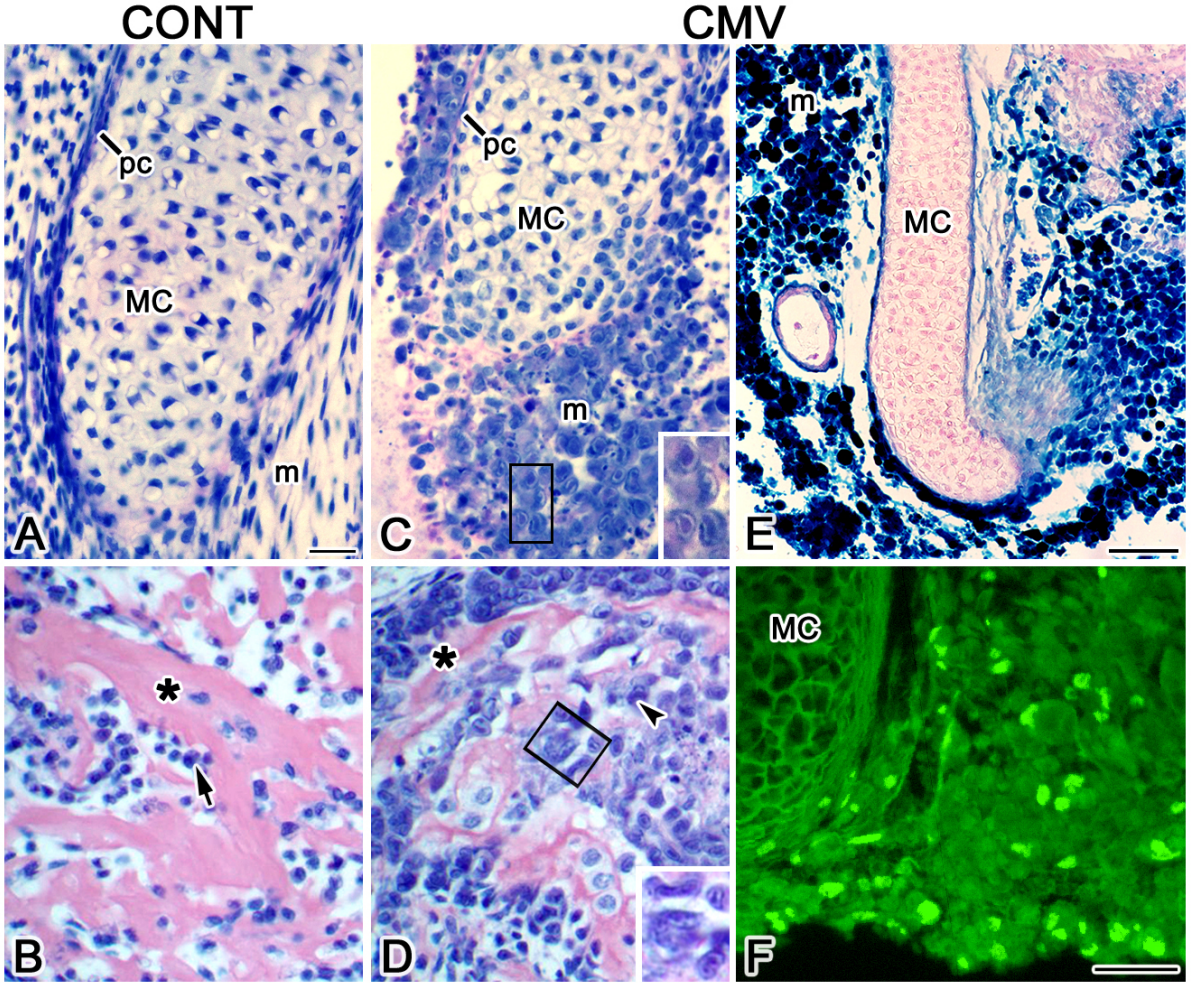
Histopathology and viral expression. A, C. Histological analysis of Meckel's cartilage. B, D. Histological analysis of mandibular bone. E. β-galactosidase staining of *lacZ*-tagged mCMV. F. Viral IE1 protein localization. In controls (A), Meckel's cartilage (MC) is composed of aligned chondrocytes bordered by a distinct perichondrial layer (pc) and is surrounded by undifferentiated mesenchyme (m). In contrast, mCMV-infected Meckel's cartilage (C) is shorter and developmentally-delayed, with misaligned chondrocytes and a disorganized perichondrium being seen. Large basophilic, pleiomorphic mesenchymal cells, some with inclusion bodies (B insert), surround the ventral and lateral regions of Meckel's cartilage and are in the perichondrium. B, D. Mandibular bone. In controls (B), the ossifying mandibular bone is characterized by matrix-depositing osteoblasts (arrow) and acidophilic trabecular osteoid (*). mCMV-infected bone (D) is dysmorphic and less ossified, with a marked decrease in acidophilic trabecular osteoid (*). Abnormal osteoblasts, some with viral inclusion bodies (arrowhead, D insert) are seen. E-F. Viral expression. β-galactosidase staining of *lacZ*-tagged mCMV (E) and viral IE1 protein localization (F) indicates mCMV infection in mesenchymal and perichondrial cells. Bar, A-D: 20 μm; C, D insert: 5 μm; E: 30 μm; F: 20 μm.

To determine the effect of active mCMV infection on embryonic MAN morphogenesis, E11 mandibular processes (MANs) were cultured in the presence or absence of 100,000 PFU/ml *lacZ*-labeled mCMV for 24 hrs [11] and then in control medium for an additional 2–15 days using a chemically-defined organ culture system [10,11,18,27]. We chose E11 MANs since no visible chondroprogenitor and osteoprogenitor mesenchymal condensations are seen in these primordia [18]. MCMV infection very early on negatively affects MAN morphogenesis, inducing a significant ~30% (P < 0.0001) decrease in size on day 3 (E11 + 3) of culture compared to controls; this significant ~30% significant size reduction persists on day 6 (E11 + 6) (P < 0.0001) and day 10 (E11 + 10) (P < 0.0001) of culture. The MAN hypoplasia (micrognathia) is consequent to reduced chondrogenesis and osteogenesis, and associated with microglossia (compare Figs. 1C, D to 1A, B and 2C to 2A).

Normally, after 10 days in culture (E11 + 10), Meckel's cartilage displays the characteristic 'wishbone-like' cartilaginous structure completely stained with Alcian blue, mandibular bone is weakly stained with Alizarin red and the mandibular condyle (a secondary cartilage) is stained with Alcian blue (Fig. 1A). In contrast, mCMV-infected E11 + 10 MANs exhibit skeletal defects. Although a cartilaginous 'wishbone-like' Meckel's cartilage is seen, a substantially smaller, dysmorphic cartilage with reduced Alcian blue staining is seen (Fig. 1C). We also find a notable decrease in Alizarin red-stained mandibular bone and Alcian blue-stained mandibular condyle (Fig. 1C).

Histological analysis demonstrates that viral infection interrupts chondrogenesis and ossification, resulting in a bent Meckel's cartilage with misaligned chondrocytes, a disorganized periochondrium, less mandibular bone ossification as indicated by a marked decrease in acidophilic trabecular bone matrix (osteoid), and reduced condylar development (compare Figs. 1D to 1B, 2C to 2A, 2D to 2B). The major cytological differences between mCMV-infected and control E11 + 10 MANs are found in mesenchymal stromal cells which are predominantly derived from cranial neural crest [12-14]. MCMV-infected MANs are characterized by stromal hypercellularity and altered mesenchymal cytology (Figs. 1D, 2C). Clusters of large basophilic, pleiomorphic cells which are hyperchromatic and exhibit increased nuclear-to-cytoplasm ratios are found in Meckel's cartilage perichondrium and in the peripherally-localized stromal cells surrounding Meckel's cartilage, mandibular bone and condyle (Figs. 1D, 2C). This aberrant cellular phenotype is also seen in mandibular osteoblasts and periostial cells (compare Fig. 2D to 2B).

To identify which cells are infected by mCMV, we determined the cell-specific localization of viral inclusion bodies (Figs. 2C, D), *lacZ*-tagged mCMV (β-galactosidase) expression (Fig. 2E) and viral immediate early protein 1 (IE1) (Fig. 2F). MCMV infection is found in stromal cells, Meckel's cartilage perichondrial cells, and mandibular osteoblasts and periostial cells. In contrast, mCMV is absent from all cartilages (Meckel's and condylar) and oral epithelium (data not shown).

**Figure 3:**
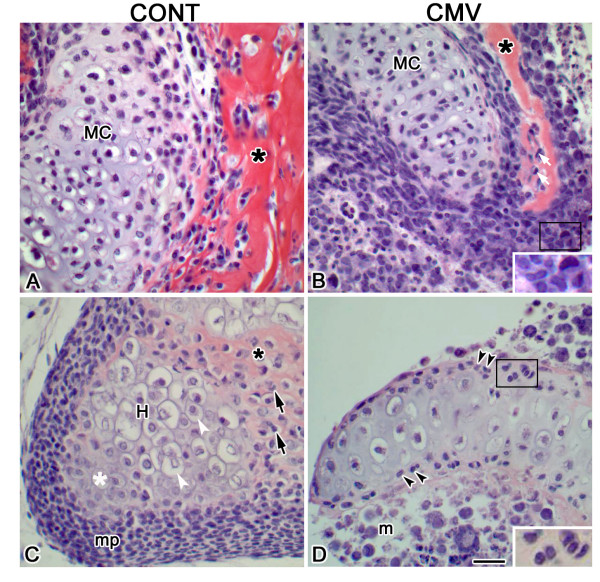
mCMV-induced Meckel's cartilage, mandibular bone and condylar defects in E11 + 16 MANs. A, B. Histologic analysis of Meckel's cartilage and mandibular intramembranous bone. The mCMV-infected MAN (B) is characterized by a notably smaller, dysmorphic Meckel's cartilage (MC) surrounded by infected perichondrial cells and a smaller, abnormal mandibular bone with decreased acidophilic trabecular bony matrix (*) compared to control (A). With mCMV infection, Meckel's cartilage and mandibular bone are surrounded by pleiomorphic, basophilic stromal cells. Viral inclusion bodies, found in stromal cells (B insert), osteoblasts (white arrows) and perichondrial cells, indicate infection. C, D. Histologic analysis of mandibular condyle. The control condylar cartilage (C) has formed a growth plate-like structure with characteristic zonation: a mesenchymal/progenitor cell layer (mp), a flattened chondrocyte zone (white *), a zone of hypertrophying (H) chondrocytes (white arrowhead), and an erosion zone composed of invading osteoblasts (black arrows) and deposited bony matrix (*). The mCMV-infected condyle (D) is abnormal, substantially smaller and atrophic, with a notable reduction in bony trabeculae and an increase in cartilaginous matrix being seen. The mesenchymal stroma (m) is composed of large, pleiomorphic and basophilic cells exhibiting atypical morphology and increased nuclear-to-cytoplasm ratio. The presence of viral infection in perichondrial/periostial (double arrowheads, D insert) and stromal cells is indicated by inclusion bodies. Bar, A-D: 20 μm; B, D insert: 10 μm.

By day 16 of culture (E11 + 16), chondrogenesis and osteogenesis has progressed in both control and mCMV-infected MANs (Fig. 3), with the smaller, abnormal Meckel's cartilage and mandibular bone phenotypes persisting in the presence of viral infection (compare Fig. 3B to 3A). In addition, mCMV infection results in a dysmorphic mandibular condyle, a secondary cartilage which undergoes endochondral ossification [28]. As shown in Figure 3C, the control E11 + 16 condylar process has formed a growth plate-like structure characterized by the zonation typical of endochondral ossification: mesenchymal/progenitor cell layer, a flattened chondrocyte layer, a zone of hypertrophying chondrocytes and an erosion zone comprised of an acidophilic trabecular bony matrix secreted by invading osteoblasts [29-32]. In contrast, the mCMV-infected condyle is smaller and atrophic, with the typical zonation being absent and a marked reduction in endochondral ossification (compare Fig. 3D to 3C). The chondroid region consists of dysmorphic chondroblasts/chondrocytes, with less distinct cell-to-cell borders and increased matrix. The cellular stroma is composed of large, pleiomorphic and basophilic affected and virally-infected (with inclusion bodies) cells which exhibit atypical morphology and increased nuclear-to-cytoplasm ratios. In some areas, the abnormal affected and infected stromal cells appear to invade the perichondrial/periosteal layer, migrating into the mineralized tissue to form osteoblasts.

### mCMV-induced changes in cell signaling proteins

Gene targeting studies have conclusively demonstrated that multiple growth and transcription factors belonging to several signaling families, including MSX, TGF-β, BMP, FGF, Hedgehog (Hh) and Wnt, regulate mandibular morphogenesis and the differentiation of its skeletal elements (see reviews, [20,33,34]). To begin to identify the mechanism underlying mCMV-induced mandibular pathogenesis, we investigated the cell-specific *in situ *protein expression and localization of sentinel components of cell signaling pathways altered by mCMV infection in embryonic salivary glands and teeth [10,11] or involved in Meckel's cartilage and mandibular bone morphogenesis.

**Figure 4:**
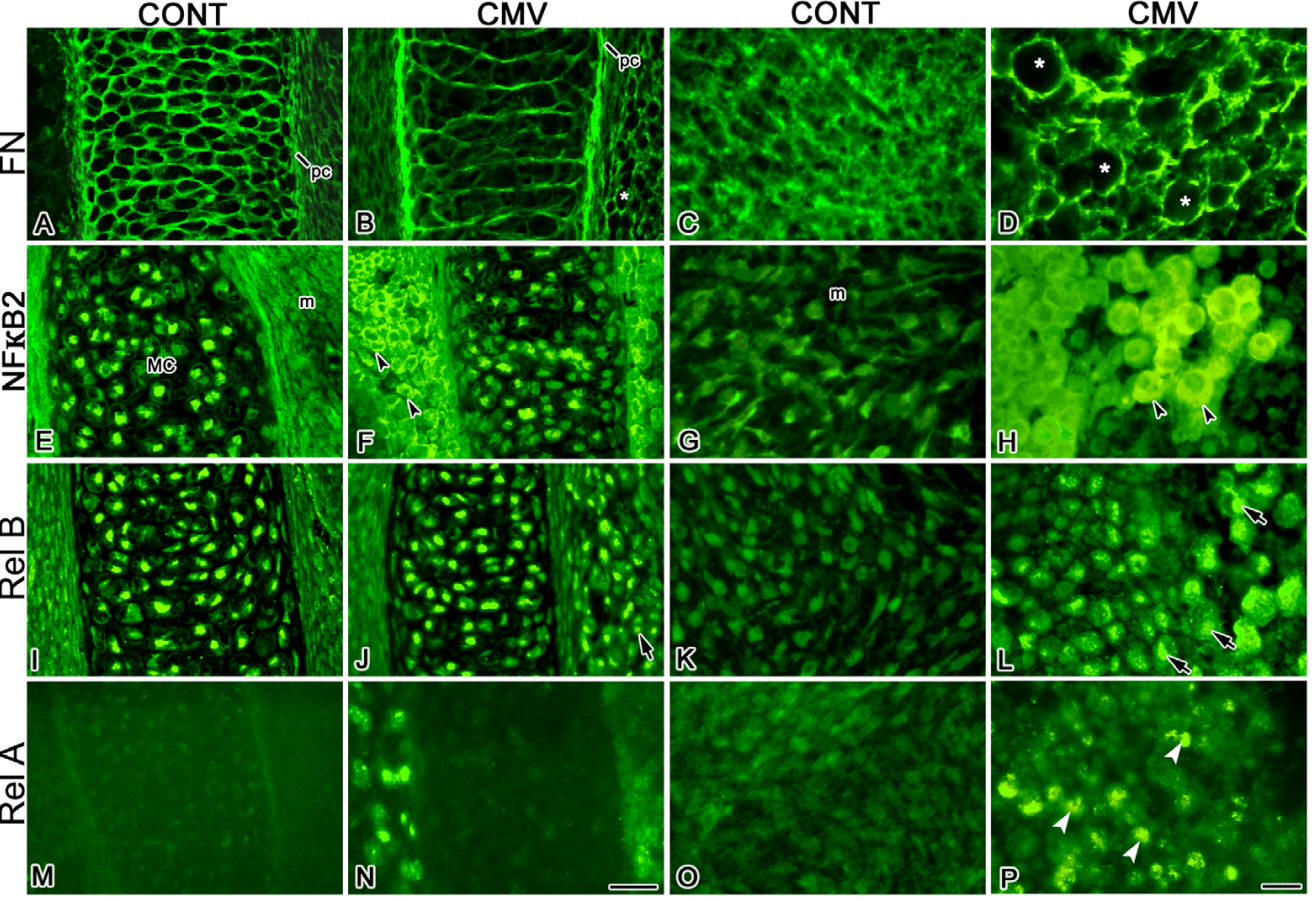
mCMV infection altered the cell-specific localization of FN, NFκ-B2, RelB, and RelA proteins in E11 + 10 MANs. A-D. FN distribution. In controls (A, C), FN is normally seen on Meckel's cartilage chondroblasts, more weakly in the perichondrium (pc) and diffusely distributed throughout the ECM. With mCMV infection (B, D), FN is intensely localized in Meckel's cartilage perichondrium (pc) and more weakly on misaligned chondrocytes. Note that FN also surrounds individual cytomegalic mesenchymal stromal cells (*). E-H. NFκ-B2 distribution. In control (E) and mCMV-infected (F) MANs, nuclear-localized NFκ-B2 is seen in Meckel's cartilage chondrocytes; NFκ-B2 is absent from control mesenchymal (m) stromal cells. mCMV infection (F, H) induced *de novo *expression of cytoplasmically-localized NFκ-B2 in abnormal stromal cells (black arrowheads). I-L. RelB distribution. A similar RelB nuclear localization is found in Meckel's cartilage chondrocytes in control (I) and mCMV-infected (J) MANs. With viral infection (J, L), *de novo *expression of nuclear-localized RelB is seen in cytomegalic stromal cells (arrows). M-P. RelA distribution. RelA is not detected in Meckel's cartilages in control (M) and mCMV-infected (N) MANs; it is also absent from control mesenchymal stroma (M, O). mCMV-infected explants (N, P) exhibit *de novo *expression of nuclear-localized RelA (white arrowheads) in abnormal stromal cells. Bar, A-B, E-F, I-J, M-N: 30 μm; C-D, G-H, K-L, O-P: 10 μm.

Fibronectin (FN) is an extracellular matrix (ECM) component shown to play important roles in craniofacial cartilage and bone formation (see reviews, [16,35]). FN acts as an adhesion molecule to mediate skeletal progenitor mesenchymal cell condensations which are essential for chondrogenesis and osteogenesis, as well as an extracellular molecule involved in regulating cell-extracellular interactions. Prior to signs of mesenchymal cell aggregation in the mandibular process, fibronectin deposition is seen in a nonrandom pattern resembling the future Meckel's cartilage primordium [36]. Given our observations that mCMV infection induced notable changes in the spatial distribution of FN in embryonic mouse salivary glands and mandibular first molars [10,11], also mandibular arch derivatives, we compared the cell-specific localization of FN in mCMV-infected and uninfected (control) E11 + 10 MANs. In controls, FN is seen on Meckel's cartilage chondroblasts in a honeycomb pattern and more weakly in the perichondrium (Fig. 4A); it is also diffusely distributed throughout the ECM (Fig. 4C). In contrast, mCMV-infected MANs exhibit intense FN immunostain in Meckel's cartilage perichondrium and more weakly on misaligned chondrocytes (compare Fig. 4B to 4A). The most dramatic difference is the aberrant localization of FN surrounding individual cytomegalic stromal cells (compare Figs. 4B, D to 4A, C). This abnormal localization pattern likely interrupts cell-cell and cell-matrix interaction required for normal morphogenesis and differentiation.

### mCMV and NF-κB expression

Spatiotemporally regulated NF-κB signaling is important for normal differentiation [37-40]. Since CMV infection induces the canonical (NF-κB1/RelA; NF-κB1/RelB) and noncanonical (NF-κB2/RelB) NF-κB pathways [10,11,41-47], we postulated that mCMV infection of E11 MANs would induce changes in NF-κB protein expression and localization. To address this question, we compared the cell-specific localization of components of the NF-κB pathways (NF-κB2, RelB and RelA) in mCMV-infected and uninfected E11 + 10 MANs (Figs. 4E–L). In controls, NF-κB2 and RelB exhibit a similar nuclear localization in Meckel's cartilage chondrocytes (Figs. 4E, I), with no substantial differences in NF-κB2 and RelB protein expression being seen with mCMV infection (compare Figs. 4F to 4E, 4J to 4I). Of particular interest is our observation that mCMV-induced *de novo *expression of both NF-κB2 (compare Figs. 4F, H to 4E, G) and RelB (compare Figs. 4J, L to 4I, K) proteins in the abnormal, cytomegalic stromal cells surrounding Meckel's cartilage and those more peripherally-located.

To determine if mCMV induced changes in the canonical pathway, we compared the pattern of RelA(p65) localization in E11 + 10 mCMV-infected MANs to controls (Figs. 4M–P). Although RelA is not detected in Meckel's cartilage in control and mCMV-infected MANs (Figs. 4M, N), mCMV infection induced a d*e novo *expression in stromal cell nuclei similar to that seen for RelB (compare Figs. 4N, P to 4J, L). Since nuclear localization indicates NF-κB or Rel activation, the nuclear localization of NF-κB2 and RelB in the Meckel's cartilage chondrocytes, as well as the absence of RelA, in both control and mCMV-infected MANs suggest that the noncanonical NF-κB pathway may play an important regulatory role during Meckel's cartilage morphogenesis. Moreover, our observation of nuclear-localized RelA and RelB, but cytoplasmically-localized NF-κB2, in cytomegalic stromal cells suggests that the canonical (NF-κB1/RelA; NF-κB1/RelB) pathway may mediate mCMV-induced MAN pathogenesis.

### mCMV and Shh signaling

**Figure 5:**
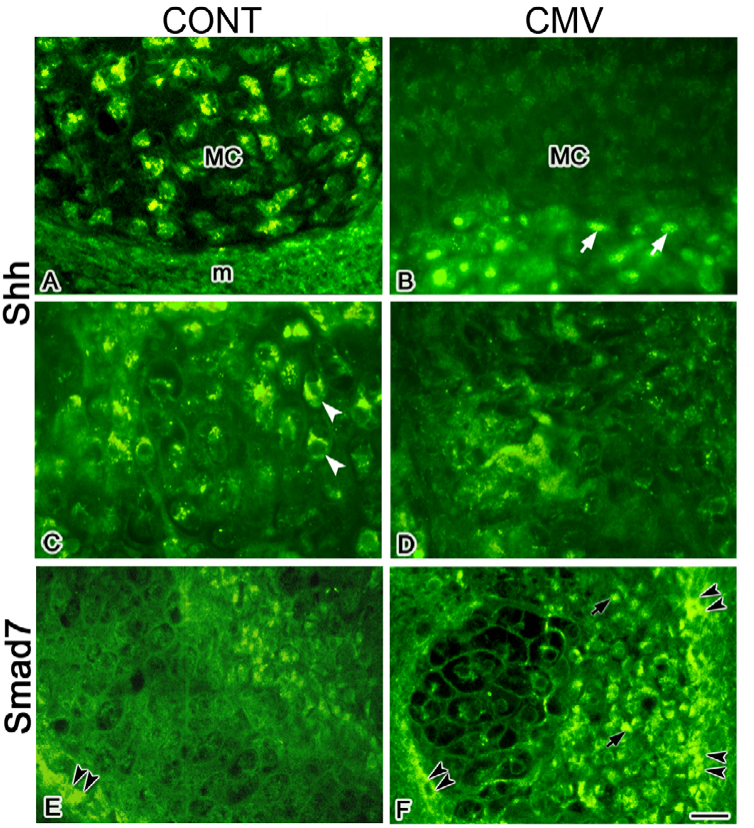
mCMV infection induced a marked increase in immunodetectable Shh and Smad7 in E11 + 10 MANs. A, B. Shh expression in Meckel's cartilage. In controls (A), Shh protein is seen in Meckel's cartilage (MC) chondrocytes but not in the perichondrium or surrounding mesenchymal stroma (m). In contrast, mCMV-infected explants (B) exhibit *de novo *expression of Shh protein in abnormal, cytomegalic stromal cells (white arrows) surrounding Meckel's cartilage but is absent from Meckel's cartilage chondrocytes. C, D. Shh expression in mandibular bone. In controls (C), Shh is seen in mandibular bone (white arrowheads). In contrast, there is a marked decrease in Shh protein in mCMV-infected bone (D). E-F. Smad7 expression in mandibular bone. In controls (E), Smad7 is primarily localized in mandibular bone periostial cells (black double arrowheads). With mCMV infection (F), there is a substantial increase in immunodetectable Smad7 in mandibular osteoblasts (black arrows) and periostial cells (black arrowheads). Bar, A-F: 20 μm.

Members of the hedgehog (Hh) family, including sonic hedgehog (Shh) and Indian hedgehog (Ihh), play pivotal roles during mandibular morphogenesis and the development of its skeletal elements [18,32,48-51]. The observation of mandibular aplasia in E15.5 and older *Shh *null mice indicates that Shh is essential for mandibular development [18]. Shh has also been shown to promote NCM differentiation into chondrocytes [51]. In addition, since *Shh *is a positively-regulated RelA response gene [52] and CMV upregulates RelA expression [10,46,47], it is reasonable to postulate that that mCMV infection would stimulate changes in Shh protein expression. Thus, we compared the cell-specific distribution of Shh protein in mCMV-infected and uninfected E11 + 10 MANs and identified notable differences (compare Figs. 5B, D to 5A, C). With mCMV infection, Shh is seen in cytomegalic stromal cells surrounding Meckel's (Fig. 5B) and condylar (data not shown) cartilages, as well as in the periphery, but is absent from both cartilages (Fig. 5B; data not shown). In contrast, Shh is found in control Meckel's (Fig. 5A) and condylar chondrocytes (data not shown) but not in surrounding and peripherally-localized stromal cells. MCMV infection also induced a substantial reduction in immunodetectable Shh protein in mandibular bone compared to control (compare Fig. 5D to 5C).

### mCMV and TGF-β signaling

It is well established that TGF-β signaling regulates MAN morphogenesis and skeletogenesis [19,53-55]. Given that (1) overexpression of Smad7, a TGF-β inhibitor, severely reduces Meckel's cartilage formation [19], (2) there is a considerable phenotypic similarity between mCMV-infected mandibular processes and those overexpressing Smad7 *in vitro *[19]; (3) CMV upregulates NF-κB1/RelA [10,42,43,46,47] and (4) NF-κB1/RelA signaling upregulates Smad7 expression [56-58], we postulated that mCMV would induce changes in Smad7 expression in developing MANs. To address this question, we compared the distribution of Smad7 in mCMV-infected and uninfected E11 + 10 MANs and demonstrate notable differences (compare Fig. 5F to 5E). There is a substantial increase in Smad7 protein in mCMV-infected mandibular osteoblasts and periosteal cells compared to control. Our results suggest that the NF-κB/Smad7 mediated inhibition of the TGFβ pathway may be important to the mCMV-induced mandibular pathology

## Discussion

Active mCMV infection of E11 *undifferentiated *mandibular processes (MANs) *in vitro *induced mandibular hypoplasia consequent to decreased Meckel's cartilage chondrogenesis and mandibular osteogenesis. The mCMV-infected MANs exhibit developmentally-delayed, misshapen Meckel's cartilages with misaligned chondrocytes and disorganized perichondria; decreased mandibular bone ossification and condylar formation; and abnormal stromal cellularity. Since MAN mesenchyme and skeletal elements are primarily derived from cranial neural crest cells [12-14], our analysis of viral distribution indicates that mCMV primarily infects NCM cells and derivatives (i.e. stromal cells, Meckel's cartilage perichondrium, mandibular bone and condylar osteoblasts/periostium). These results are consistent with our prior observations in other first branchial arch derivatives (salivary glands and teeth) that mCMV has a particular tropism for neural crest-derived mesenchymal cells [10,11]. The absence of mCMV infection in abnormal Meckel's and condylar cartilages and a subset of atypical mesenchymal cells suggests that, as in mCMV-infected glands and molars, paracrine factors likely mediate the viral effect on surrounding uninfected cells. Furthermore, perichondrial cells surrounding cartilages synthesize factors which regulate cell proliferation and differentiation during chondrogenesis [54,59]. Thus, it is likely that mCMV-induced cartilaginous defects are due to changes in essential signaling factors secreted by virally-infected perichondrial cells. Moreover, since perichondrial cells are infected, it is unclear why mCMV did not infect Meckel's cartilage and condylar chondrocytes even though they are also derived from cranial neural crest cells. One possible explanation is that the biochemical program of chondrocytes is not permissive for mCMV infection and replication.

Chondrogenesis and osteogenesis are complex processes which involve several different phases, including chondroprogentior or osteoprogenitor mesenchymal cell condensation and overt differentiation of chondroblasts, chondrocytes and osteoblasts (see reviews, [16,35]). Condensation, the earliest sign of the initiation of skeletogenesis, is a critical step for both chondrogenesis and osteogenesis which occurs when a previously dispersed population of mesenchymal cells forms an aggregation (condensation). Following condensation, cell-cell communications and interactions with ECM molecules are required for subsequent skeletogenesis. Thus, the ECM functions as both an adhesive molecule during initial condensation and a mediator of cell-matrix interactions during chondrogenesis and osteogenesis.

FN, an ECM component, has been shown to mediate mesenchymal cell condensation and cellular interactions with the extracellular environment (see reviews, [16,60]). In the embryonic MAN, FN is first deposited in a nonrandom pattern resembling the Meckel's cartilage to be [36] and then on Meckel's cartilage chondroblasts, chondrocytes and perichondrium [36]. In the present study, FN is localized in control MANs on Meckel's cartilage chondrocytes, weakly in the perichondrium, and diffusely throughout the ECM (Figs. 4A, C). MCMV infection induces a substantial change in FN expression so that FN surrounds individual infected and affected neural crest-derived aberrant stromal cells (Fig. 4D). A similar abnormal FN distribution is also seen in mCMV-infected neural crest-derived salivary gland stroma and dental papilla mesenchyme [10,11]. Since FN plays an important role for mesenchymal cell condensation and cell-matrix interactions, our data suggests that aberrant FN localization interferes with interactions necessary for normal skeletogenesis.

NF-κB belongs to a family of transcription factors composed of NF-κB1 (p50), NF-κB2 (p52), RelA(p65), RelB and c-Rel. They form homo- and hetero-dimers to activate a wide variety of genes involved in many biological functions (see reviews, [61-63]). Since NF-κB1 and NF-κB2 lack a transactivation domain, they can only be activated and translocated into the nucleus when dimerized with RelA, RelB or c-Rel. The IκB family of inhibitory proteins keeps inactive NF-κB/Rel dimers in the cytoplasm. Degradation of IκB allows the NF-κB/Rel complex to translocate to the nucleus, and bind to NF-κB and/or Rel recognition sites to regulate gene transcription. Two NF-κB pathways, the canonical NF-κB1/Rel pathway and noncanonical NF-κB2/RelB pathways, have been identified and their functions investigated (see reviews, [61-63]).

It is well established that CMV infection induces the canonical and noncanonical NF-κB pathways in fibroblasts and other cell types [41-46], which in turn facilitates viral replication [41-44]. We have recently demonstrated that mCMV-induced embryonic salivary gland and tooth pathogenesis is centered around canonical and noncanonical NF-κB activation in NCM cells [10,11].

Previous studies have demonstrated that NF-κB/Rel plays a role during chondrogenesis and osteogenesis [64-66]. Since nuclear localization is indicative of NF-κB/Rel activation, our observation of *nuclear*-localized NF-κB2 and RelB in uninfected (control) Meckel's cartilage chondrocytes (Figs. 4E, I) suggests that the noncanonical NF-κB2/RelB pathway participates in normal Meckel's cartilage chondrogenesis. Moreover the *de novo *appearance of *nuclear*-localized RelA and RelB (but *not *NF-κB2) in mCMV-infected stromal cells (Figs. 4H, L, P) suggests that the canonical NF-κB1/RelA (or RelB) is also important to mandibular pathogenesis in a way similar to that seen in salivary glands and teeth, namely the induction of abnormal paracrine signaling [10,11].

The TGF-β family of growth factors regulates a wide range of biological functions during organogenesis, including cell proliferation, differentiation and ECM formation (see review, [67]). *In vivo *and *in vitro *loss-of-function studies have demonstrated that TGF-β signaling plays key roles during mandibular chondrogenesis and osteogenesis [19,53-55]. In the present study, we found a notable increase in immunodetectable Smad7, an inhibitor of TGF-β signaling, in the smaller, developmentally-delayed mCMV-infected mandibular bone (Figs. 5E, F), suggesting that Smad7-mediated inhibition of TGF-β signaling also contributes to mCMV-induced mandibular pathogenesis.

Loss-of-function mutant mouse studies have clearly established that members of the Hh family play pivotal roles during mandibular morphogenesis and the development of cartilage and bone elements [18,32,48-50]. Of particular note here is the observation of mandibular aplasia in E15.5 and older *Shh *null mice [18,48]. Although E13.5 *Shh *null mice present a small mesenchymal condensation in the region of a presumptive Meckel's cartilage in the hypoplastic MANs, by E15.5 *Shh *mutants exhibit a mere remnant of the MAN with no evidence of Meckel's cartilage differentiation [18]. Presently, we find that mCMV-infected hypoplastic MANs exhibit aberrant Shh protein expression (Figs. 5B, D). Shh, normally seen in Meckel's cartilage chondrocytes, is found in abnormal, cytomegalic stromal cells but not in Meckel's cartilage chondrocytes. There is also a marked decrease in immunodetectable Shh in mandibular bone osteoblasts and periostium. Thus, mCMV-induced MAN dysplasia may also be due, in part, to reduced Shh expression in Meckel's cartilage and mandibular bone, as well as aberrant localization in stromal cells.

## Conclusion

MCMV infection of *undifferentiated *embryonic mouse MANs *in vitro *induced mandibular hypoplasia (micrognathia) and abnormal skeletal elements. Specifically, mCMV infection of primarily neural crest-derived mesenchymal cells resulted in aberrant stromal cellularity and induced smaller, dysplastic Meckel's cartilages, mandibular bones and condyles due to interrupted chondrogenesis and osteogenesis. Initial localization studies suggest that the pathogenesis is centered around the canonical and noncanonical NF-κB pathways, and there is an unusual juxtaposition of abnormal stromal cells and surrounding matrix. The absence of mCMV infection in abnormal cartilages and a subset of abnormal mesenchymal cells suggests that, as in mCMV-infected glands and molars, paracrine factors likely mediate the viral effect on surrounding uninfected cells. Finally, since it is critically important for normal development that signaling molecules be expressed in appropriate cell populations, the aberrant localization of components of key signaling pathways may reveal the pathogenic mechanism underlying mandibular malformations. That both Shh and Smad7 are NF-κB/Rel response genes provides a good starting point for a wider genomic and proteomic investigation of the pathogenesis.

## Methods

### Embryonic culture system and mCMV infection

Female B10A/SnSg mice, obtained from Jackson Laboratories (Bar Harbor, ME), were maintained and mated as previously described [11]; plug day = day 0 of gestation. Timed-pregnant females were sacrificed on gestation day 11 (E11) and embryos were dissected in cold phosphate-buffered saline (PBS). All animal studies were conducted with the approval of the appropriate committees regulating animal research. An Animal Review Board and a Vivaria Advisory Committee review all applications to ensure ethical and humane treatment. E11 mandibular processes (MANs) were cultured using a modified Trowell method as previously described [18]. The defined media consisted of BGJb (Invitrogen Corporation, Carlsbad, CA) supplemented with 0.5 mg ascorbic acid/ml and 50 units/ml penicillin/streptomycin (Invitrogen Corporation), pH 7.2; media is changed daily.

#### mCMV infection

on day 0, MANs were incubated in the presence or absence of 100,000 plaque-forming units (PFU)/ml of *lacZ*-tagged mCMV RM427^+ ^[68] for 24 hrs and then cultured in virus-free BGJb defined media for an additional 2–15 (E11 + 3 to E11 + 16) days. Explants were collected and processed for whole mount morphology, routine histology, or immunolocalization.

### Whole mount and histological analyses

For whole mount morphological and size analyses, MANs were photographed using a Wilde dissecting microscope at 25 × and the area determined using Image-Pro Version 4.0 (Media Cybernetics, Silver Spring, Maryland). The following groups were analyzed: E11 + 3 [Cont (n = 17); mCMV-infected (n = 16)]; E11 + 6 [Cont (n = 18); mCMV-infected (n = 17)]; E11 + 10 [Cont (n = 15); mCMV-infected (n = 12)]. The significance of area differences between viral-infected and control explants were determined by Student t-test. To assay skeletal development, E11 + 6 and E11 + 10 mCMV-infected or control whole mounts were stained with Alcian blue which stains for cartilage and alizarin red which stains for bone as previously described [18]. For histological analyses, explants were fixed for 4 hrs in Carnoy's fixative at 4°C or overnight in 10% neutral buffered formalin at room temperature, embedded in paraffin, serially-sectioned at 8 μm and stained with hematoxylin and eosin as previously described [11,18]. For each experimental protocol, 3–18 primordia were analyzed.

### mCMV analysis

To obtain a measure of mCMV infection, we assayed for distribution of β-galactosidase (*lacZ*) activity and viral immediate early (IE1) proteins as previously described [11]. **β-galactosidase (β-gal) staining**: E11 + 6 and E11 + 10 mCMV-infected explants were fixed, stained and photographed as previously described [11]. Whole mounts were then dehydrated through graded alcohols, embedded in paraffin, serially-sectioned at 8 μm and counterstained with eosin. **IE1 distribution**: E11 + 10 explants were fixed in Carnoy's fixative, serially-sectioned at 8 μm, and incubated overnight with anti-IEI as previously described [11,18]. Controls consisted of sections incubated with mouse IgG alone. For each experimental protocol, 3 mCMV-infected explants were analyzed.

### Antibodies and immunostaining

Immunolocalization was conducted essentially as previously described [10,11,27,69] using the following polyclonal (Pab) antibodies: RelB (SC-30887), NFκB p52 (SC-298), Shh (SC-9024), Smad 7 (SC-11392) (Santa Cruz Biotechnology, Santa Cruz, CA); FN (F3648, Sigma-Aldrich Corp., St. Louis, MO); NFκB p65(RelA) (Ab-435) (ABM Inc, Vancouver, CA). Nuclei were counterstained with DAPI (Invitrogen Corporation).

## Authors' contributions

TJ and MM conceived and designed the study. TJ was involved in and coordinated all experiments, and drafted the manuscript. MM participated in analysis of histopathology and localization data and helped draft the manuscript. GA participated in morphological and immunofluorescent experiments and generated all figures. PS participated in histopathology analysis. PB participated in histopathology and immunolocalization analyses. All authors read and approved the final manuscript.
